# Plasma neurofilament light admission levels and development of axonal pathology in mild traumatic brain injury

**DOI:** 10.1186/s12883-023-03284-6

**Published:** 2023-08-15

**Authors:** Iftakher Hossain, Mehrbod Mohammadian, Henna-Riikka Maanpää, Riikka S. K. Takala, Olli Tenovuo, Mark van Gils, Peter Hutchinson, David K. Menon, Virginia F. Newcombe, Jussi Tallus, Jussi Hirvonen, Timo Roine, Timo Kurki, Kaj Blennow, Henrik Zetterberg, Jussi P. Posti

**Affiliations:** 1 Department of Neurosurgery, Neurocenter, Turku University Hospital, Turku, Finland; 2 Turku Brain Injury Center, Turku University Hospital, Turku, Finland; 3 Department of Clinical Neurosciences, University of Turku, Turku, Finland; 4 Department of Clinical Neurosciences, Neurosurgery Unit, University of Cambridge, Addenbrooke’s Hospital, Cambridge, UK; 5 Intensive Care Medicine and Pain Management, Perioperative Services, Turku University Hospital and University of Turku, Turku, Finland; 6 Faculty of Medicine and Health Technology, Tampere University, Tampere, Finland; 7 Division of Anaesthesia, University of Cambridge, Addenbrooke’s Hospital, Cambridge, UK; 8 Department of Radiology, University of Turku and Turku University Hospital, Turku, Finland; 9 Turku Brain and Mind Center, University of Turku, Turku, Finland; 10 Department of Neuroscience and Biomedical Engineering, Aalto University School of Science, Turku, Finland; 11 Department of Psychiatry and Neurochemistry, Institute of Neuroscience and Physiology, The Sahlgrenska Academy at the University of Gothenburg, Mölndal, Sweden; 12 Clinical Neurochemistry Laboratory, Sahlgrenska University Hospital, Mölndal, Sweden; 13 Department of Molecular Neuroscience, UCL Institute of Neurology, Queen Square, London, UK; 14 UK Dementia Research Institute at UCL, University College London, London, UK; 15 Hong Kong Center for Neurodegenerative Diseases, Hong Kong, China

**Keywords:** Neurofilament light protein, Diffusion tensor imaging, Diffusion-weighted magnetic resonance imaging

## Abstract

**Background:**

It is known that blood levels of neurofilament light (NF-L) and diffusion-weighted magnetic resonance imaging (DW-MRI) are both associated with outcome of patients with mild traumatic brain injury (mTBI). Here, we sought to examine the association between admission levels of plasma NF-L and white matter (WM) integrity in post-acute stage DW-MRI in patients with mTBI.

**Methods:**

Ninety-three patients with mTBI (GCS ≥ 13), blood sample for NF-L within 24 h of admission, and DW-MRI ≥ 90 days post-injury (median = 229) were included. Mean fractional anisotropy (FA), mean diffusivity (MD), axial diffusivity (AD), and radial diffusivity (RD) were calculated from the skeletonized WM tracts of the whole brain. Outcome was assessed using the Extended Glasgow Outcome Scale (GOSE) at the time of imaging. Patients were divided into CT-positive and -negative, and complete (GOSE = 8) and incomplete recovery (GOSE < 8) groups.

**Results:**

The levels of NF-L and FA correlated negatively in the whole cohort (*p* = 0.002), in CT-positive patients (*p* = 0.016), and in those with incomplete recovery (*p* = 0.005). The same groups showed a positive correlation with mean MD, AD, and RD (*p* < 0.001—*p* = 0.011). In CT-negative patients or in patients with full recovery, significant correlations were not found.

**Conclusion:**

In patients with mTBI, the significant correlation between NF-L levels at admission and diffusion tensor imaging (DTI) measurements of diffuse axonal injury (DAI) over more than 3 months suggests that the early levels of plasma NF-L may associate with the presence of DAI at a later phase of TBI.

**Supplementary Information:**

The online version contains supplementary material available at 10.1186/s12883-023-03284-6.

## Introduction

Mild traumatic brain injury (mTBI), which includes concussion, accounts for 80% – 90% of all TBIs presenting to emergency departments [1]. At a cellular level, the pathophysiology of mTBI consists primarily of diffuse injury caused by stretching and tearing of the brain tissue, followed by a complex cascade of neurometabolic changes [2–5]. Diffuse axonal injury (DAI) is the main form of diffuse injury, and results from acceleration / deceleration forces leading to axonal shearing [6, 7]. Computed tomography (CT), the most commonly used imaging method for acute TBI, is generally unable to detect DAI [8, 9]. Also, conventional MRI is poor in showing or quantifying DAI, and neuropathological examination is the only accurate method for diagnosing DAI at the moment [7, 10, 11]. Advanced neuroimaging methods, such as diffusion-weighted (DW) magnetic resonance (MR) imaging, have been shown to be sensitive enough to detect small abnormalities associated with DAI [12–14]. Diffusion tensor imaging (DTI) [15, 16] is a technique to evaluate DAI in patients with mTBI in the subacute and chronic phases [15, 17–20], but is still considered mainly as a research tool. Fractional anisotropy (FA) and mean diffusivity (MD) have been the main focus in DTI studies after an mTBI [20]. DW-MRI based structural connectivity after mTBI has been recently shown to be related to outcome [21].

Regrettably, biomarkers to assess the degree of axonal injury or the multidimensional pathophysiological events following mTBI are not yet available for clinical use [22–24]. Neurofilament light (NF-L) protein is an axonal biomarker that can be measured in blood samples with ultrasensitive Single molecule array (Simoa) technology [25–27]. NF-L is mainly expressed in the long myelinated WM axons [2, 27, 28]. A significant association between DTI measures of DAI and the serum levels of NF-L following severe TBI (sTBI) has been reported, suggesting that the levels of NF-L may reflect the degree of axonal injury [27, 29]. Elevated levels of plasma NF-L in mTBI have been found in contact sports athletes, although those studies did not report the correlation between the levels of NF-L and WM integrity [5, 30]. Recently, a significant association between the early plasma levels of NF-L and the outcome in patients with mTBI has been reported in a prospectively collected well-characterized cohort by our research group [31], which supports the concept that NF-L is a potential blood biomarker to explore the complex pathophysiology of axonal injury following mTBI. A recent study by Shahim et al. examined the time course and diagnostic utility of NF-L in subacute and chronic TBI, demonstrating that increased serum concentrations of NF-L at enrolment correlated with the DTI measures of DAI [32]. Another multicenter prospective study also reported that the levels of plasma NF-L reflect the WM damage following TBI [33]. A recent pilot study on the adolescent soccer players also reported a significant association between DTI metrics and proteomic blood biomarkers, including NF-L [34].

Since it is known that blood levels of NF-L and DW-MRI are both associated with outcome of patients with mTBI, we sought to investigate the possible association between the admission levels of plasma NF-L and WM integrity, measured using post-acute DTI metrics. The hypothesis of this study is that acute level of plasma NF-L following mTBI may help clinicians better stratify those patients who require further DTI imaging to understand acquired axonal injury.

## Methods

### Study population

This prospective study was part of the EU-funded TBIcare (Evidence-based Diagnostic and Treatment Planning Solution for Traumatic Brain Injuries) project. From November 2011 to October 2013, 93 patients with mTBI [Glasgow Coma Scale (GCS) ≥ 13] and a control group of 21patients with orthopedic injury (OI) were recruited, with blood samples available within 24 h from the arrival to the emergency department (ED) of the Turku University Hospital, Finland.

The inclusion criteria for patients with mTBI were: lowest GCS ≥ 13, age ≥ 18 years, clinical diagnosis of TBI, and indications for acute head CT according to NICE criteria (http://www.nice.org.uk/guidance/cg176). The exclusion criteria were: age < 18 years, blast-induced or penetrating injury, chronic subdural hematoma, inability to live independently due to pre-existing brain disease, admission more than 2 weeks from the injury, not living in the district (thereby preventing follow-up visits), not speaking native language, or no consent received.

The inclusion criteria for patients with OI were: age ≥ 18 years, acute nontrivial OI, no concomitant TBI, and no CNS involvement. The exclusion criteria were: any suspicion of concomitant acute TBI, history of any brain disease or TBI, need for admission to intensive care due to polytrauma, or trivial injuries with no necessity for emergency measures or follow-up.

### Analysis of NF-L

Although majority of the samples were obtained within 24 h of admission, they were not always drawn within 24 h after injury. All samples were kept in cold ice and processed within 1 h and stored at − 80 °C until analysis. At the day of the measurements, samples are thawed and kept on ice until diluted into sample diluent according to the protocol provided in the kit insert. NF-L is a stable analyte that is not sensitive to storage temperature or repeated freezing–thawing [35]. Plasma NF-L levels were measured using the Human Neurology 4-Plex A assay on an HD-1 Simoa instrument according to instructions from the manufacturer (Quanterix, Billerica, MA). The measurements were performed in one round of experiments using one batch of reagents by board-certified laboratory technicians who were blinded to clinical data. Quality control (QC) samples were analyzed in each run, with coefficients of variations of 4.4% at 13.9 pg/mL and 6.1% at 7.1 pg/mL for NF-L. The lower limit of detection (LLoD) and the lower limit of quantification (LloQ) for NF-L were 0.104 pg/mL and 0.241 pg/mL, respectively and a calibration range between 0.533 pg/mL and 453.0 pg/mL.

### TBI severity and outcome grading

For the assessment of TBI severity, the lowest recorded GCS assessed by paramedics at the scene of accident or during transport, and / or by an emergency physician at the time of admission was used[31]. The overall injury severity of the patients was assessed using the Injury Severity Score (ISS) [36]. The descriptive system proposed by Marshall et al. was used to classify the CT scans, where class 1 corresponds with normal CT, classes 2 – 4 with diffuse injuries, and classes 5 – 6 CTs with mass lesions [37]. Patients were divided into CT-positive and -negative groups based on presence or absence of intra-cranial injury.

### Outcome

The outcome was assessed between 4 – 16 months from the injury using the Extended Glasgow Outcome Score (GOSE), and in close proximity (same day or within a few days) to the DTI scan [38]. Outcomes were dichotomized to complete recovery (GOSE = 8), or incomplete recovery (GOSE < 8). Every patient was evaluated by the same experienced neurologist at the Turku University Hospital.

### MRI acquisition

The MRIs were acquired at Turku University Hospital with a Siemens Verio 3 T scanner. Fluid attenuated inversion recovery, Susceptibility-Weighted imaging, T2-weighted, and DW-MR images were obtained from each subject. DW-MRI utilizing spin-echo, echo-planar imaging was obtained using the following parameters: TR 11.7 s, TE 106 ms, voxel size of 2 × 2 × 2 mm. Diffusion gradients were applied in 64 directions with a b-value of 1000 s/mm^2^. FA and MD of DW-MRI were used as indicators of WM integrity at a later stage. However, axial diffusivity (AD) and radial diffusivity (RD) metrics were also taken into consideration.

### DTI analyses

DW-MR images were corrected for subject’s motion, eddy current, and EPI distortions [39, 40]. Tensors were then fitted in each voxel and anisotropy and diffusivity maps were calculated using the ExploreDTI tool [41]. ExploreDTI was used to perform the pre-processing of the DW-MR images as images with reverse phase encoding were not acquired in this study and the tensor estimation was done in ExploreDTI to avoid probable errors in flipping of gradient orientations. FA, MD, AD, and RD maps were then fed into FMRIB Software Library (FSL). After data were pre-processed, FA images from each subject were non-linearly aligned to the FAMRIB_FA template in MNI space and were projected to a skeletonized mean FA image using tract-based spatial statistics [42]. Similarly, MD, AD, and RD images were projected to the WM skeleton using the non-linear warps and skeleton projection performed in the previous step for FA images. Mean DTI metrics values were then calculated from the whole skeletonized WM tracts of the whole brain.

### Statistical analyses

All statistical analyses were performed in IBM SPSS (Version 24, Armonk, NY, USA) and MATLAB (R2018b, Natick, MA, USA). Normality of the variables were assessed using Shapiro–Wilk test and histogram analysis. Non-parametric Mann–Whitney U test was used to compare the levels of NF-L between patient groups. Generalized linear modelling, with age and sex as covariates, was performed to assess the difference in WM microstructural properties between patient groups. Correlations between the levels of NF-L and DTI metrics (FA, MD, AD, and RD) in different patient groups/subgroups were analyzed with (partial) Spearman’s rank correlation coefficient (ρ) accounting for age and sex. The above-mentioned methods were also utilized to compare the levels of NF-L between patients with mTBI and patients with OI. A confidence interval of 95% was used to specify the significance of the results.

## Results

Patient characteristics are described in detail in Table 1. Ninety-three patients with mTBI were dichotomized to overlapping radiological and clinical outcome groups of CT-positive (*n* = 40, 43.0%) or CT-negative (*n* = 53, 57%), and with complete (*n* = 35, 37.6%) or incomplete (*n* = 58, 62.4%) recovery. We also performed a separate analysis on CT-negative patients with complete (*n* = 29, 54.7%) or incomplete (*n* = 24, 45.3%) recovery.

**Table 1:** Patient Demographics and Clinical Characteristics

	All mTBI	CT-negative	CT-positive	*p*-value	Complete recovery	Incomplete recovery	*p*-value	OI controls	*p*-value
No. of patients (%)	93*	53* (57.0)	40 (43.0)		35*	58*		21	
Years of Age				0.111 ^a^			0.791^a^		0.870^a^
Median (IQR)	47.00 (36)	46.0 (34)	52.00 (43)		47.00 (44)	47.00 (31)		43 (29)	
Mean (SD)	45.99 (19.59)	43.17 (18.4)	49.73 (20.72) (20.72)(20.72)		45.26 (21.91)	46.43 (18.24)		45.24 (16.1)	
Sex n (%)				0.043 ^b^			0.178^b^		0.305^b^
Male	64 (68.8)	32 (60.4)	32 (80.0)		27 (77.1)	37 (63.8)		12 (57.1)	
Female	29 (31.2)	21 (39.6)	8 (20.0)		8 (22.9)	21 (36.3)		9 (42.9)	
Worst GCS n (%)				0.106 ^c^			0.775^c^		0.004^c^
15	62 (66.7)	40 (75.5)	22 (55.0)		22 (62.))	40 (69.0)		21 (100)	
14	25 (26.9)	10 (18.9)	15 (37.5)		11 (31.4)	14 (24.1)		0	
13	6 (6.5)	3 (5.7)	3 (7.5)		2 (5.7)	4 (6.9)		0	
Cause of injury n (%)				0.031 ^c^			0.399^c^		0.057^c^
Road traffic crash	28 (30.1)	17 (32.1)	11 (27.5)		7 (20.0)	21 (36.2)		4 (19.0)	
Incidental fall	49 (52.7)	22 (41.5)	27 (67.5)		22 (62.9)	27 (46.6)		12 (57.1)	
Violence/assault	9 (9.7)	8 (15.1)	1 (2.5)		4 (11.4)	5 (8.6)		0	
Other non-intentional injury	4 (4.3)	4 (7.5)	0		2 (5.7)	2 (3.4)		5 (23.8)	
Suicide attempt	1 (1.1)	1 (1.9)	0		0	1 (1.7)		0	
Other	2 (2.2)	1 (1.9)	1 (2.5)		0	2 (3.4)		0	
Isolated TBI n (%)	51 (54.8)	32 (60.4)	19 (47.5)	0.217 ^b^	22 (62.9)	29 (50.0)	0.227^b^	-	-
Extracranial injuries with TBI n (%)	42 (45.2)	21 (39.6)	21 (52.5)		13 (37.1)	29 (50.0)		-	-
CT findings (Marshall Grade), n (%)							0.333^c^		-
Diffuse injury I, no visual pathology	53 (57.0)	53 (100)	0		24 (68.6)	29 (50.0)		-	
Diffuse injury II	25 (26.9)	0	25 (62.5)		8 (22.9)	17 (29.3)		-	
Diffuse injury III	3 (3.2)	0	3 (7.5)		0	3 (5.2)		-	
Diffuse injury IV	2 (2.2)	0	2 (5.0)		1 (2.9)	1 (1.7)		-	
Evacuated mass lesions	6 (6.5)	0	6 (15.0)		2 (5.7)	4 (6.9)		-	
Non-evacuated mass lesions	4 (4.3)	0	4 (10.0)		0	4 (6.9)		-	
GOSE n (%)				0.086^c^			0.000^c^		-
8	35 (37.6)	24 (45.3)	11 (27.5)		35 (100)	0		-	
7	32 (34.4)	16 (30.2)	16 (40.0)			32 (55.2)		-	
6	13 (14.0)	9 (17.0)	4 (10.0)			13 (22.4)		-	
5	4 (4.3)	2 (3.8)	2 (5.0)			4 (6.9)		-	
4	5 (5.4)	2 (3.8)	3 (7.5)			5 (8.6)		-	
3	4 (4.3)	0	4 (10.0)			4 (6.9)		-	
Injury Severity Score				0.001^d^			0.037^d^		0.001^d^
Median (IQR)	11.00 (15)	6.00 (11)	13.50 (9)		6.00 (12.00)	11.00 (13.00)		4.00 (0)	
Mean (SD)	12.12 (9.88)	9.55 (9.218)	15.40 (0.83)		9.56 (9.05)	13.65 (10.11)		4.57 (2.89)	
Admitted to hospital	68 (73.1)	32 (60.4)	36 (90.0)	0.001^b^	24 (31.4)	44 (75.9)	0.442^b^	17 (81.0)	0.457^b^
Discharged from the emergency department	25 (26.9)	21 (39.6)	4 (10.0)		11 (68.6)	14 (24.1)		4 (19.0)	

Computed tomography negative (CT-negative) = Marshall 1, Computed tomography positive (CT-positive) = Marshall 2–6. Glasgow Outcome Scale Extended (GOSE) 8 = complete recovery, Glasgow Outcome Scale Extended (GOSE) 1–7 = incomplete recovery

*For the injury severity score (ISS), there was no information available for 2 patients in the TBI cohort

^a^Independent samples T-test

^b^Chi-Square

^c^Fisher's Exact Test and

^d^Mann–Whitney U-test all with 0.05 significance level

The majority (*n* = 80, 86.5%) of the blood samples from mTBI patients was obtained within 24 h of the hospital admission. The exact injury time was available for 60.2% (*n* = 56) of the subjects with a median time elapse from injury to blood sampling of 11 h (IQR = 13.8). Among patients for whom the exact injury time was unavailable, 8 patients were sampled within 24 h and 29 patients were sampled after 24 h from the injury. DW-MRI was obtained 126 – 429 days after the injury (median = 229, IQR = 71). Injury severity score (ISS) was higher in the CT-positive group (median = 13.5, IQR = 9) than in the CT-negative group (median = 6, IQR = 11, *p* = 0.001). Further, differences were found between patients with complete recovery (median = 6, IQR = 12) and incomplete recovery (median = 11, IQR = 13, *p* = 0.037). There was a male predominance in the whole mTBI cohort, but this was even more pronounced in the CT-positive group (80.0%, *p* = 0.043).

### Differences in DTI metrics in patients with mTBI

**Fig. 1:**
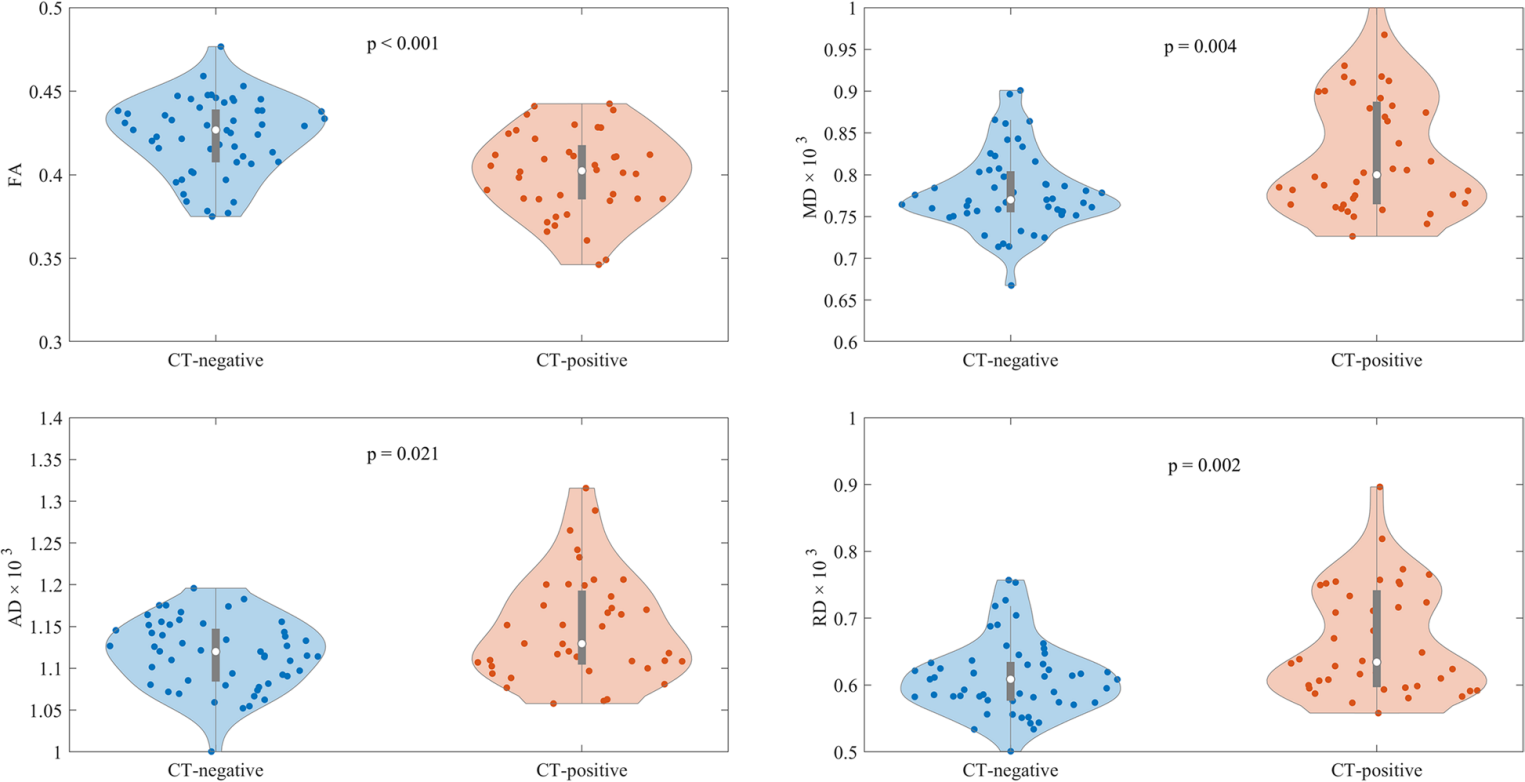
Diffusion tensor imaging (DTI) metrics values for the different mild traumatic brain injury (mTBI) subgroups. Computed tomography positive = CT-positive, computed tomography negative = CT-negative, Glasgow Outcome Scale Extended (GOSE) 8 = complete recovery, and Glasgow Outcome Scale Extended (GOSE) 1–7 = incomplete recovery. FA = fractional anisotropy, MD = mean diffusivity, AD = axial diffusivity, RD = radial diffusivity

The results for DTI metrics are presented in Fig. 1 and Table 2. FA values were higher in CT- negative patients (mean = 0.423, SD = 0.023) than in CT-positive patients (mean = 0.401, SD = 0.025) (*p* < 0.001). MD levels were higher in the CT-positive subgroup (mean = 0.827, SD = 0.073) than in the CT-negative subgroup (mean = 0.78, SD = 0.046) (*p* = 0.004). AD in the CT-positive subgroup (mean = 1.15, SD = 0.063) was higher than in the CT-negative subgroup (mean = 1.12, SD = 0.04) (*p* = 0.021). RD was also higher in the CT-positive subgroup (mean = 0.666, SD = 0.081) than in the CT-negative subgroup (mean = 0.612, SD = 0.055) (*p* = 0.002). Between the subgroups of complete and incomplete recovery, there were no differences in the various DTI metrics (Table 2).

**Table 2:** Comparison of neurofilament light levels between mild traumatic brain injury patients and orthopedic injury controls. The comparison between the diffusion metrics of the mTBI subgroup

	NF-L levels		FA		MD (× 10^–3^)		AD (× 10^–3^)		RD (× 10^–3^)	
Group	median [IQR]	*p*-value^a^	mean [SD]	p-value^b^	mean [SD]	*p*-value^b^	mean [SD]	*p*-value^b^	mean [SD]	*p*-value^b^
all mTBI	14.28 [27.32]	0.038	0.414 [0.026]	-	0.8 [0.064]	-	1.13 [0.053]	-	0.635 [0.072]	-
CT-Negative	10.42 [8.3]	0.890	0.423 [0.023]	*p* < 0.001	0.78 [0.046]	0.004	1.12 [0.04]	0.021	0.612 [0.055]	0.002
CT-Positive	31.37 [48.54]	*p* < 0.001	0.401 [0.025]		0.827 [0.073]		1.15 [0.063]		0.666 [0.081]	
Complete recovery	11.16 [10.68]	0.383	0.417 [0.022]	0.145	0.792 [0.049]	0.19	1.12 [0.44]	0.291	0.625 [0.058]	0.171
Incomplete recovery	16.22 [40.97]	0.01	0.411 [0.028]		0.8 [0.071]		1.13 [0.059]		0.641 [0.08]	
Orthopedic Injury Controls	10.8 [6.8]	-	-	-	-	-	-	-	-	-

^a^Mann-Whitney U test, *p*-values show the difference between Orthopedic Injury Controls and the patient groups

^b^Generalized linear model where age and sex are used as confounders, *p*-values represent the comparison between patient groups only

*mTBI* mild traumatic brain injury, *CT* Computed tomography, *NF-L* Neurofilament light, *FA* Fractional anisotropy, *MD* Mean diffusivity, *AD* Axial diffusivity, *RD* Radial diffusivity, *IQR* Interquartile range, *SD* Standard deviation

### Correlation between NF-L levels and DTI metrics in patients with mTBI

A negative correlation was observed between the level of NF-L and FA in the whole mTBI group (ρ = -0.323, *p* = 0.002), in CT-positive patients (*ρ* = -0.389, p = 0.016) and in patients with incomplete recovery (*ρ* = -0.367, *p* = 0.005) (Table 3). In the complete recovery or CT-negative subgroups, no correlation was observed (Table 3). No correlation was detected in CT-negative patients with incomplete or complete recovery, either (Table 3).

**Table 3:** Correlation between admission neurofilament light (NF-L) levels and diffusion measures (adjusted for age and sex)

		FA		MD		AD		RD	
Group	Number of patients	Spearman’s rho	*p*-value	Spearman’s rho	*p*-value	Spearman’s rho	*p*-value	Spearman’s rho	*p*-value
all mTBI	93	-0.323	0.002	0.343	*p* < 0.001	0.313	0.003	0.324	0.002
CT-Negative	53	-0.032	0.825	0.194	0.172	0.214	0.132	0.163	0.252
CT-Positive	40	-0.389	0.016	0.408	0.011	0.453	0.004	0.4	0.013
Complete recovery	35	-0.184	0.306	0.182	0.311	0.044	0.808	0.179	0.318
Incomplete recovery	58	-0.367	0.005	0.395	0.003	0.394	0.003	0.35	0.008
CT-Negative with complete recovery	29	-0.131	0.562	0.22	0.325	0.131	0.561	0.223	0.318
CT-Negative with incomplete recovery	24	0.032	0.873	0.078	0.699	0.216	0.278	0.060	0.766

Computed tomography negative (CT-negative) = Marshall 1, Computed tomography positive (CT-positive) = Marshall 2–6. Glasgow Outcome Scale Extended (GOSE) 8 = complete recovery, Glasgow Outcome Scale Extended (GOSE) 1–7 = incomplete recovery. *FA* Fractional anisotropy, *MD* Mean diffusivity, *AD* Axial diffusivity, *RD* Radial diffusivity

A positive correlation was observed between the levels of NF-L and MD in the whole mTBI cohort (*ρ* = 0.343, *p* < 0.001), in CT-positive patients (*ρ* = 0.408, *p* = 0.011), and in patients with incomplete recovery (*ρ* = 0.395, *p* = 0.003) (Table 3). No correlations were observed in the subgroups of all CT-negative patients, in all patients with complete recovery, or in CT-negative patients with incomplete or complete recovery (Table 3).

The levels of NF-L showed a positive correlation with AD in all patients with mTBI (*ρ* = 0.313, p = 0.003) (Table 3). The subgroup analysis revealed a positive correlation also in CT-positive patients (ρ = 0.453, *p* = 0.004), and in patients with incomplete recovery (*ρ* = 0.394, *p* = 0.003), but no correlation was found between NF-L levels and AD in patients with complete recovery or in the CT-negative patients (Table 3). Again, no correlation was observed in CT-negative patients with either incomplete or complete recovery (Table 3).

Like other diffusivity measures, RD was positively correlated with NF-L levels in all patients with mTBI (*ρ* = 0.324, *p* = 0.002), as well as in CT-positive (*ρ* = 0.4, *p* = 0.013) and incomplete recovery (*ρ* = 0.35, *p* = 0.008) groups, but not in patients with complete recovery, in CT-negative patients, or in the subgroups of CT-negative patients with incomplete or complete recovery (Table 3).

Patients with posttraumatic amnesia (PTA) 24 h or less (*n* = 50) were analyzed separately. None of the correlations between NF-L levels and DTI metrics were significant in those patients even when divided into CT-positive and CT-negative subgroups (Supplementary table 1).

### OI controls

There were 21 patients with orthopedic extracranial injuries in the control group. Median age for control subjects was 43 (IQR = 29) and most were male 12 (57.1%). The most common injury types were ankle fractures n = 12 (54.5%) and wrist fractures *n* = 2 (9.1%). Compared to the control group (median = 10.8, IQR = 6.8), the levels of NF-L were higher in the whole mTBI cohort (median = 14.28, IQR = 27.32, *p* = 0.038), in the subgroup of CT-positive patients (median = 31.37, IQR = 48.54, *p* < 0.001), and in patients with incomplete recovery (median = 16.22, IQR = 40.97) (*p* = 0.01), but not in the CT-negative subgroup or in subjects with complete recovery (Table 2).

### Differences in NF-L levels in patients with mTBI

**Fig. 2:**
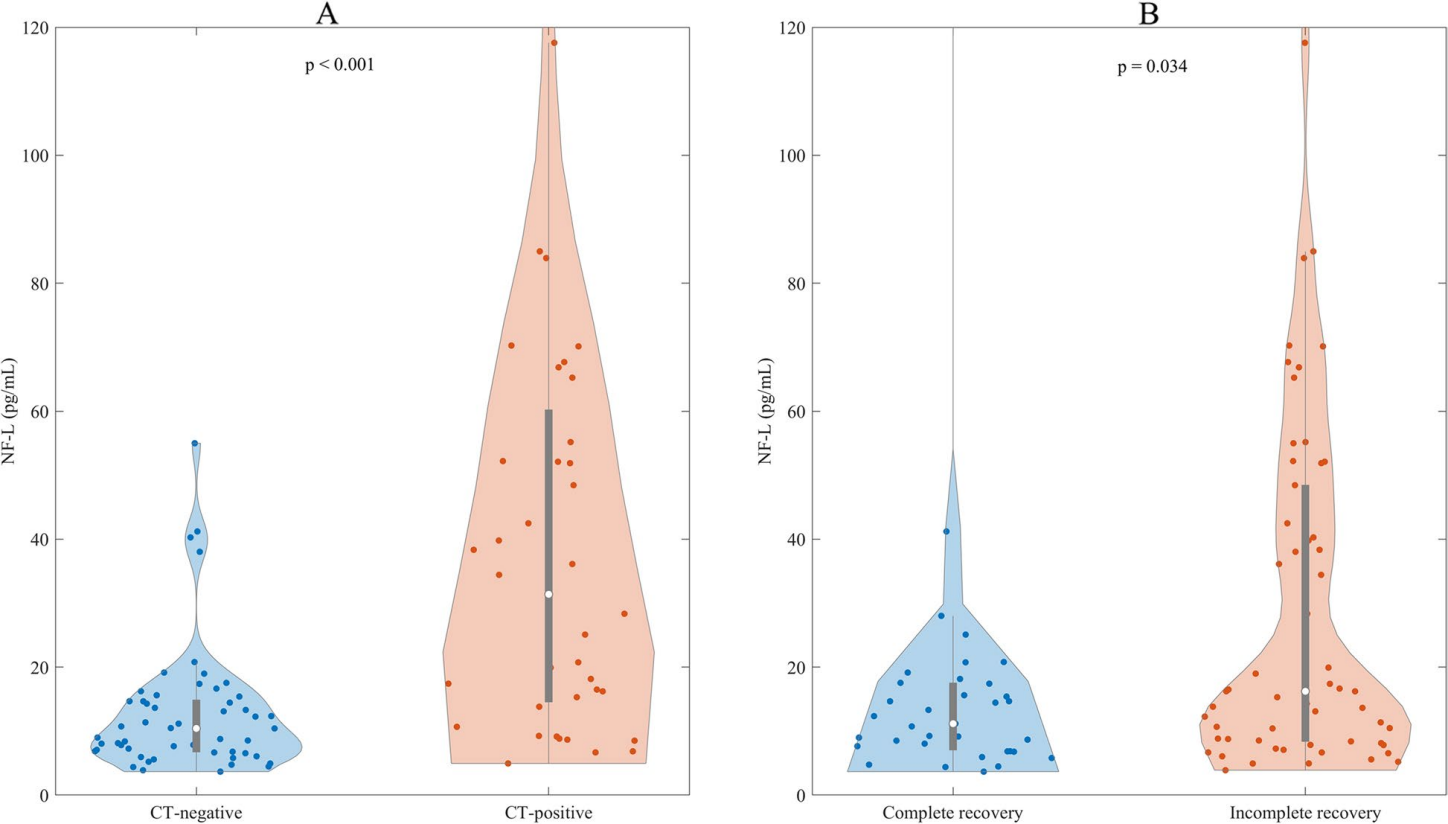
Levels of NF-L in patients dichotomized based on CT findings (**A**) or their outcome (**B**). Computed tomography positive = CT-positive, computed tomography negative = CT-negative, Glasgow Outcome Scale Extended (GOSE) 8 = complete recovery, and Glasgow Outcome Scale Extended (GOSE) 1–7 = incomplete recovery

The levels of NF-L were higher in CT-positive patients (median = 31.37, IQR = 48.54) compared with CT-negative patients (median = 10.42, IQR = 8.31) (*p* < 0.001). Further, patients with incomplete recovery had higher NF-L levels (median = 16.22, IQR = 40.97) compared with patients with complete recovery (median = 11.16, IQR = 10.68) (*p* = 0.034) (Fig. 2).

## Discussion

This prospective, observational study of patients with mTBI investigated the association between the admission levels of plasma NF-L with WM integrity, measured using DTI metrics from DW-MR images more than 3 months from the injury. Moreover, we also compared the admission levels of NF-L between the patients with mTBI and the OI control group. The main findings were as follows: (1) Significant negative correlations between the levels of NF-L and FA and significant positive correlations between the levels of NF-L and the other diffusivity measures were observed in the whole mTBI cohort, in patients with CT-positive findings, and in patients with incomplete recovery. (2) The admission levels of NF-L were significantly higher in the whole mTBI cohort, in patients with CT-positive findings, and in patients with incomplete recovery, compared to the control group. (3) Lower anisotropy and higher diffusivity measures were observed in CT-positive patients compared with patients without any CT findings. (4) Admission levels of NF-L were higher in CT-positive patients and in patients with incomplete recovery compared with CT-negative patients and patients with complete recovery respectively.

NF-L protein has been extensively studied as a potential body fluid biomarker to investigate the ongoing axonal injury following TBI [33, 34, 57]. Several studies have shown that patients with mTBI or concussion had significantly higher levels of NF-L compared to healthy individuals or orthopedic controls, not only in the acute phase following the injury, but also in the subacute and chronic phases [32, 43–45]. After TBI, a significant increase in serum levels of NF-L has been observed, which persisted up to 10 – 12 days after injury [46]. In addition, the admission levels, as well as the levels at several time-points, were correlated with the outcome of TBI [46]. The levels of NF-L have been shown to be significantly elevated in contact sports athletes, for example, professional hockey players who suffered from symptoms after repetitive mTBI [5, 44]. It has also been reported that a single mild to moderate TBI may cause long-term neuroaxonal degeneration, which could be detected by NF-L as a surrogate marker [32]. Of note, our research group lately reported that the levels of NF-L were able to differentiate patients with complete recovery from incomplete recovery, and favorable outcome from unfavorable outcome after mTBI. These results applied not only to the whole cohort, but also to patients with CT-positive mTBI, and the early levels of NF-L strongly correlated with outcome [31]. Recent studies utilizing the admission and late samples reported that serum levels of NF-L were longitudinally associated with DTI estimates of DAI [32]. Lower anisotropy and higher diffusivity measures may suggest compromised axonal integrity, demyelination, Wallerian degeneration and overall, might be an indication of axonal degeneration following mTBI [47–49]. These findings are in accordance with previous studies in patients with TBI [50–53]. It has been reported that the elevated blood levels of NF-L at 6 months was significantly related to the metrics of microstructural injury on DTI [54]. A recent multicenter prospective study of advanced fluid and imaging markers of axonal injury after moderate to severe TBI, BIO-AX-TBI [55], demonstrated that the levels of plasma NF-L and DTI metrics are closely related in quantifying underlying axonal injury subacutely after TBI. In this study, microdialysate taken directly from damaged WM was found to contain very high levels of NF-L and this concentration of NF-L in microdialysis fluid significantly correlated with the levels of NF-L in plasma. Moreover, in the same study, the plasma levels of NF-L also correlated with histopathologically defined axonal injury within the WM, which was produced by an experimental injury model [33]. Thus, the association between the plasma levels of NF-L and DTI metrics indicates that plasma NF-L measurement may reflect the damage of WM of the brain following TBI. The results of the present study are thus consistent with the aforementioned studies.

The kinetics of NF-L as a blood biomarker has been recently explored by using several time points of sampling following TBI. These studies found that the peak of NF-L is between 10 days and 6 weeks following injury and that subacute levels strongly correlated with outcome [33, 56]. These results are in agreement with the concept that DAI is a slow, long-lasting process, as suggested by longitudinal imaging studies [57–61]. In the current study, only admission samples were used, since few patients with mTBI had samples available from later days. The observed correlation between the admission levels of NF-L and DTI metrics probably reflects the consequences of rapid regional axonal damage in those who show visible traumatic lesions, rather than reflecting the many secondary pathophysiological cascades contributing to subsequently evolving WM damage. This is supported by the fact that in CT-negative patients, significant correlations were not seen.

A well-characterized, prospectively collected study population is a major strength of the study, but there are also limitations that need to be acknowledged. Although NF-L does not have sources outside the nervous system, it is known that trauma itself has at least indirect consequences on the brain. Thus, patients with orthopedic injuries were analyzed as controls in order to increase the reliability of the results. Besides the small sample size and a single-center study, other key limitations of this study are the timing of NF-L not being tight, the lack of data for NF-L levels at later timepoints after admission, and lack of DTI data at several time points to conduct longitudinal analyses. Given that NF-L is a slow marker [62], i.e., the peak in blood comes days-weeks after injury, and that one-time DTI measures are unable to describe the temporal evolution of DAI [63], this study is unable to shed light on the progression of such axonal injury. The main practical limitation, thus, is that this study is unable to show if NF-L at a later timepoint could predict incomplete recovery in patients who are CT-negative after an mTBI.

For the critical interpretation of the study findings, it is also evident that the results were driven by patients with more severe injuries – especially those who had mass lesions or multiple contusions. To partially address this, CT-positive and CT-negative findings were analyzed separately, and significant findings were found only in the CT-positive subgroup. For the CT-negative subjects there was no significant correlation between the levels of NF-L and any of the diffusion metrics in either the complete or incomplete recovery subgroups. This suggests that the correlations observed in the incomplete recovery group, including both the CT-positive and the CT-negative, have been heavily influenced by the CT-positive group.

Indeed, the severity of injury in our mTBI cohort was worse than in an average mTBI population typically seen in the ED, therefore it cannot be considered to represent cases with mTBI in general. It is important to know that we classified the patients to severity groups solely based on the admission GCS score. Classifying the severity of TBI using the lowest recorded GCS is one of the important limitations of this study. In our series, the mildest cases of mTBI were often discharged before the possibility to recruit and a relatively large percentage of our mTBI cohort showed traumatic intracranial CT abnormalities, consequently requiring hospital admission. Furthermore, even though all the recruited patients had GCS ≥ 13, categorized as mTBI, some patients had PTA for > 24 h post injury, which is an indication of higher severity of TBI according to several classifications. PTA was assessed retrospectively using the Rivermead method [64] at the outcome visit, whereas prospective evaluation is often considered to have higher reliability. Further analysis on patients with mTBI, also considering PTA, found that there was no significant correlation between the levels of NF-L and any of the diffusion metrics, irrespective of CT results. This shows that the significant results found are strongly driven by the cases at the severe end. Even though the variability of the GOSE assessment is another limitation, the same experienced blinded neurologist performed the assessments of all patients. These issues have been elaborated thoroughly in our previous publications. Functional outcome is much more complex than just complete or incomplete recovery as assessed with the GOSE and clinicians’ assessment of disability also vary and may be different from those of their patients [65].

Due to the logistics and the limited availability to scan patients, it was not possible to scan all subjects within a certain window of time after injury hence the difference in time from injury to imaging could be a limitation of this study. Acquisition of DW-MR images with a single shell and only one b0 is another shortcoming in this study. Acquiring multi-shell DW-MR data with several b0 images using advanced analysis approaches, such as neurite orientation dispersion and density imaging [66], or using novel deep learning approaches suitable for single-shell DW-MRI [67], might reveal signs of axonal injury not detectable in the current study. Furthermore, TBSS suffers from inherent limitations such as the inability to correctly differentiate complex WM fiber configurations and being susceptible to partial volume effects [68].

## Conclusion

The significant correlation between NF-L levels at admission and DTI measurements of DAI over more than 3 months suggests that plasma NF-L may associate with the presence of DAI during the acute phase of TBI and possibly help clinicians to recognize those patients who need more careful follow-up. This needs to be validated using several time-points of biomarker sampling, longitudinal DTI data, and larger cohorts. Large multicenter studies with adequate control groups, including patients with polytrauma as well as healthy controls, should be conducted before blood biomarker research findings can be translated into clinical practice. Moreover, future research should establish standard methods for quantification on different analytical platforms and define cut-off values for these blood biomarkers across different injury subtypes and age groups [57–59].

### Supplementary Information

**Additional file 1: Supplementary Table 1.** Correlation between the levels of neurofilament light and diffusion metrics in patients with mild traumatic brain injury with Glasgow Coma Scale of 13 and above and a duration of less than 24 hours of posttraumatic amnesia (PTA). FA = fractional anisotropy, MD = mean diffusivity, RD = radial diffusivity, AD = axial diffusivity.

## Data Availability

The datasets used and/or analyzed during the current study available from the corresponding author on reasonable request.
