# Supplementary material for: Plasma neurofilament light admission levels and development of axonal pathology in mild traumatic brain injury
Source: BMC Neurol. 2023 Aug 15;23:304. doi: 10.1186/s12883-023-03284-6 (PMC10426141; doi:10.1186/s12883-023-03284-6)
Supplement: Supplementary file 1 — Additional file 1: Supplementary Table 1. Correlation between the levels of neurofilament light and diffusion metrics in patients with mild traumatic brain injury with Glasgow Coma Scale of 13 and above and a duration of less than 24 hours of posttraumatic amnesia (PTA). FA = fractional anisotropy, MD = mean diffusivity, RD = radial diffusivity, AD = axial diffusivity. [file 12883_2023_3284_MOESM1_ESM.pdf]

Supplementary Table 1. Correlation between the levels of neurofilament light and diffusion metrics in patients with mild traumatic brain injury with Glasgow Coma Scale of 13 and above and a duration of less than 24 hours of posttraumatic amnesia (PTA). FA = fractional anisotropy, MD = mean diffusivity, RD = radial diffusivity, AD = axial diffusivity.

		FA		MD		AD		RD	
Group	Number of subjects	Spearman's rho	p-value	Spearman's rho	p-value	Spearman's rho	p-value	Spearman's rho	p-value
All patients with PTA ≤24 hours	50	-0.023	0.877	0.176	0.877	0.126	0.392	0.259	0.076
CT negative	34	0.146	0.424	0	0.999	-0.025	0.893	0.088	0.633
CT positive	16	0.243	0.402	- 0.017	0.953	-0.05	0.865	0.11	0.707
